# The challenge of equipoise: qualitative interviews exploring the views of health professionals and women with multiple ipsilateral breast cancer on recruitment to a surgical randomised controlled feasibility trial

**DOI:** 10.1186/s40814-022-01007-1

**Published:** 2022-02-28

**Authors:** Jenny Ingram, Lucy Beasant, John Benson, Adrian Murray Brunt, Anthony Maxwell, James Richard Harvey, Rosemary Greenwood, Nicholas Roberts, Norman Williams, Debbie Johnson, Zoe Winters

**Affiliations:** 1 Bristol Medical School, University of Bristol, Bristol, BS8 1NU UK; 2 Addenbrookes Hospital, Cambridge and School of Medicine Anglia Ruskin University, Cambridge, UK; 3 School of Medicine, Keele University, Staffordshire, UK; 4 Nightingale Centre, Wythenshawe Hospital, Manchester, UK; 5 Division of Informatics, Imaging & Data Sciences, University of Manchester, Manchester, UK; 6 Division of Molecular and Clinical Cancer Sciences, School of Medical Sciences, University of Manchester, Manchester, UK; 7 University Hospitals Bristol and Weston NHS Foundation Trust, Bristol, UK; 8 Division of Surgery and Interventional Science, University College London, London, UK

**Keywords:** Equipoise, Multiple ipsilateral breast cancer, Therapeutic mammoplasty, Mastectomy, Randomisation, Qualitative

## Abstract

**Background:**

A multicentre feasibility trial (MIAMI), comparing outcomes and quality of life of women with multiple ipsilateral breast cancer randomised to therapeutic mammoplasty or mastectomy, was conducted from September 2018 to March 2020. The MIAMI surgical trial aimed to investigate recruitment of sufficient numbers of women. Multidisciplinary teams at 10 breast care centres in the UK identified 190 with MIBC diagnosis; 20 were eligible for trial participation but after being approached only four patients were recruited. A nested qualitative study sought to understand the reasons for this lack of recruitment.

**Methods:**

Interviews were conducted from November 2019 to September 2020 with 17 staff from eight hospital-based breast care centres that recruited and attempted to recruit to MIAMI; and seven patients from four centres, comprising all patients who were recruited to the trial and some who declined to take part. Interviews were audio-recorded, anonymised and analysed using thematic methods of building codes into themes and sub-themes using the process of constant comparison.

**Results:**

Overarching themes of (1) influences on equipoise and recruitment and (2) effects of a lack of equipoise were generated. Within these themes, health professional themes described the barriers to recruitment as ‘the treatment landscape has changed’, ‘staff preferences and beliefs’ which influenced equipoise and patient advice; and how different the treatments were for patients. Patient themes of ‘altruism and timing of trial approach’, ‘influences from consultants and others’ and ‘diagnostic journey doubts’ all played a part in whether patients agreed to take part in the trial.

**Conclusions:**

Barriers to recruiting to breast cancer surgical trials can be significant, especially where there are substantial differences between the treatments being offered and a lack of equipoise communicated by healthcare professionals to patients. Patients can become overwhelmed by numerous requests for participation in research trials and inappropriate timing of trial discussions. Alternative study designs to the gold standard randomised control trial for surgical interventions may be required to provide the high-quality evidence on which to base practice.

**Trial registration:**

ISRCTN (ISRCTN17987569) registered on April 20, 2018, and ClinicalTrials.gov (NCT03514654).

## Key messages regarding feasibility

- It was uncertain whether it was possible to recruit women with multiple ipsilateral breast cancer to be randomised to one of two surgical treatment options.
- This qualitative study illustrated the challenges of running a randomised controlled feasibility trial in a discipline where the evidence base for clinical decision-making changes rapidly and can be impacted by high-profile international consensus meetings with dogmatic statements. Recruitment to breast cancer surgical trials presents significant barriers, especially where there is a substantial difference between the treatments offered and equipoise is not communicated effectively by recruiters.
- Well-designed prospective cohort studies rather than surgical RCTs may be a more pragmatic way to generate high-quality evidence on which to base best practice in an era of patient-centred care in breast cancer surgery.

## Background

The diagnosis of multiple ipsilateral breast cancer (MIBC) has increased over recent years with up to 20% of patients presenting with more than one tumour focus in the same breast [1]. The safety and efficacy of surgical treatments employing breast-conservation techniques, such as therapeutic mammoplasty (TM), compared to mastectomy remains uncertain, with no trials specifically addressing these aspects [1, 2]. Historically, mastectomy has been undertaken for 80 to 90% of MIBC, due to perceptions of higher ipsilateral breast tumour recurrence (IBTR) and poorer cosmesis, when breast conservation is employed [3]. Hence, mastectomy has usually been recommended for most patients with more than one tumour focus, whether in the same or different quadrants. However, following the St Gallen consensus conference in 2017 [4, 5], surgical centres are increasingly offering TM for MIBC, despite little clinical trial data.

Choosing between mastectomy and breast-conserving surgery (BCS) can be a difficult decision for women, involving personal preferences about body image and sexuality, as well as concerns relating to cancer recurrence [6]. Longer term follow-up of randomised controlled trials (RCTs) has confirmed the equivalence of survival outcomes for BCS compared to mastectomy for unifocal cancers [7–11], with recent evidence that BCS may be associated with an overall survival benefit [12–14]. Given these findings and the less intrusive nature of BCS compared to mastectomy, it might be assumed that women with early-stage breast cancer would be more likely to choose BCS [7, 8].

However, variation in BCS management is observed worldwide, highlighting the need for further research into the factors influencing surgical treatment decisions [15, 16]. Improvements in systemic therapies and radiotherapy techniques have contributed to dramatic reductions in IBTR rates for unifocal cancers treated with BCS (approximately 0.5% per annum) [12, 14, 17]. These adjuvant treatment improvements may also reduce the chance of in-breast recurrence when BCS is performed for MIBC [1, 2, 18]. Recent cohort studies have reported that TM is associated with fewer complications than mastectomy and immediate reconstruction for single site unilateral cancers [19]. Although significant scientific and psychosocial advances have been made in improving women’s choices for breast cancer treatment, there are still many unknown factors in the decision-making processes surrounding the surgical treatment of breast cancer [16, 20].

**Fig. 1:**
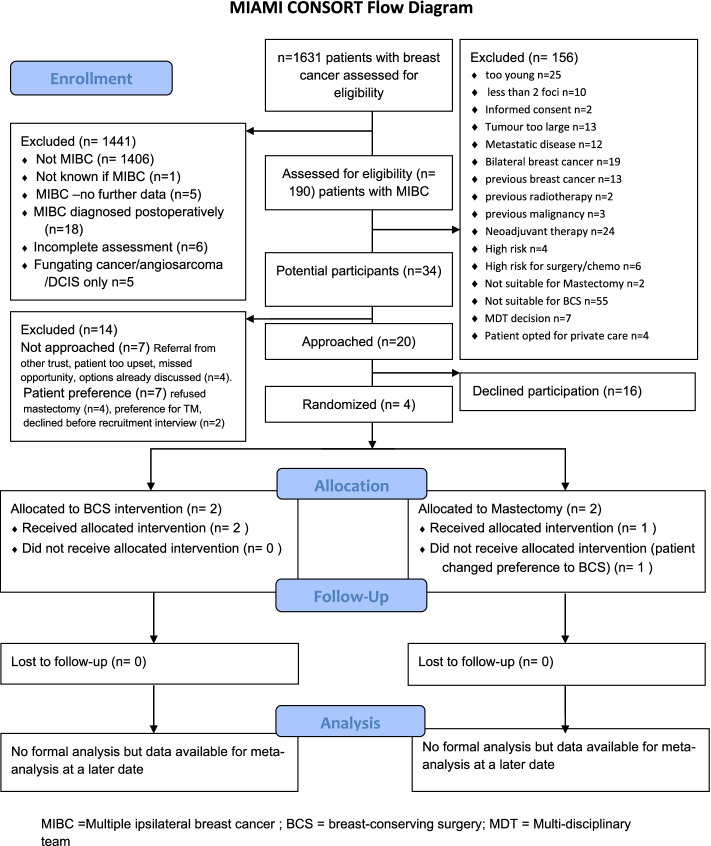
CONSORT diagram for MIAMI

A multicentre feasibility trial (MIAMI) comparing clinical outcomes and quality of life measures for women with MIBC randomised to TM or mastectomy, with or without reconstruction, has been conducted to investigate recruitment of women and acceptability of trial processes [2]. The trial aimed to recruit 50 patients from a potential pool of approximately 700 with MIBC, with suitable women approached in hospital-based breast care centres from September 2018 to March 2020. Screening women with breast cancer at local multidisciplinary team meetings (MDTs) identified 190 with MIBC diagnosis; 20 were eligible for trial participation but after being approached only four patients were recruited. Sites were not open to recruitment throughout the potential recruiting period due to staff availability and signoff delays. The main reason for all sites being closed was the IRMER (Ionising Radiation (Medical Exposure) Regulations) signoff which was initiated following trial opening and took an additional 6 months to complete. Some sites did not switch back from screening only to full recruitment, thus limiting the pool of potential recruits. Figure 1 shows the trial CONSORT diagram detailing the reasons for excluding patients and the eligible patients who declined.

A nested qualitative study sought to understand the reasons for the poor accrual by interviewing staff from sites that did and did not recruit, and also interviewing all the patients who were recruited to the trial and some who declined to take part.

## Methods

### Setting

The MIAMI feasibility trial included breast cancer patients in 10 secondary breast care centres across the UK. Patient eligibility criteria included those deemed fit for adjuvant treatments, 40 years or older, histologically confirmed MIBC (largest tumour focus up to 50mm as part of multifocal/multicentric disease sites) and surgically amenable to TM. Surgeons were discouraged from discussing surgical options outside a dedicated trial consultation setting, and research nurses addressed key trial questions using a standardised communication template with guidance in the trial consultation process.

The qualitative interviews aimed to explore the acceptability of recruitment, the concept of randomisation, type of surgery and outcome questionnaires. These issues would inform the design of the planned subsequent main trial and development of appropriate care pathways.

### Participants

All women recruited into the study were invited to participate in a semi-structured telephone interview conducted by a qualitative researcher (LB), 6–12 months after randomisation. A small opportunistic sample of women who declined to take part in the trial were also invited for interview. The patient interviews explored the recruitment processes, information given, reflections on their treatment and also being part of a trial. A purposive sample of health professionals including surgeons and research nurses from recruiting and non-recruiting sites were invited to take part in telephone interviews with the researchers (JI or LB) exploring their experience of screening and recruiting patients, barriers to approaching patients and the trial processes and treatments. Interview schedules were based on information from the literature, team discussions and Patient Advisory Group input.

### Analysis

Trained qualitative researchers (LB, JI), with extensive experience of evaluating health care services, conducted the thematic data analysis. All interviews were digitally recorded (with informed consent), transcribed verbatim, anonymised and analysed using thematic methods of building codes into themes and sub-themes using the process of constant comparison (facilitated by NVIVO 11 software: QSR International Pty Ltd). All the interviews were coded by LB and five interviews were double coded by JI to increase robustness. Codes and themes were developed and discussed at regular intervals by the qualitative group during data collection and analysis to achieve consensus [21].

Ethical approval was granted from the NHS National Research Ethics Service (NRES) Committee London—City & East (REC reference 18/LO/0133) on March 14, 2018, and HRA approval on March 15, 2018.

### Results

Eight of the 10 sites were represented in the interviews; four sites recruited one patient each (B, D, F, J). Seventeen health professionals were interviewed between November 2019 and July 2020, including 10 surgeons and seven research nurses; four were male and 13 were female (Table 1). Seven patient interviews were conducted from July to September 2020; four with the women recruited to the MIAMI trial, and three with women who declined to take part in the trial. Overall, five patients underwent TM and two had mastectomy as their definitive surgical procedures.

**Table 1:** Details of those who took part in interviews

**Health professionals interviewed**	**Recruiting sites** (*n*=4)	**Non-recruiting sites** (*n*=4)	**Total interviews** (*n*=17)
Surgeons	4	6	10
Research nurses	3	4	7
Males	1	3	4
Females	6	7	13
**Patients interviewed**	**Recruited** (*n*=4)	**Declined** (*n*=3)	
Surgical procedure	TM =3; Mastectomy =1	TM = 2; Mastectomy =1	

The overarching themes generated were grouped into (1) factors influencing equipoise and recruitment and (2) effects of a lack of equipoise. Within these themes, health professional sub-themes described the barriers to recruitment in ‘the treatment landscape has changed’; and the effects of this as **‘**staff preferences and beliefs influencing equipoise’, and how different the treatments were for patients. Patient themes influencing recruitment included ‘altruism and timing’ of trial approach, and the effects of a lack of equipoise as ‘influences from consultants and others’ and ‘diagnostic journey doubts’ which determined whether women agreed to take part in the trial. Themes are shown in Table 2 and are presented below with illustrative quotes: staff quotes are identified by role and site; patient interviews identified by site and whether recruited to the trial or not.

**Table 2:** Themes described by health professional and patients that influenced equipoise and recruitment to the trial

	Health professional themes	Patient themes
**Influences on equipoise and recruitment**	**The treatment landscape has changed**—St Gallen consensus and usual care treatment has changed.	**Altruism** (to help others)—recruited patients; **Timing**—too many trials offered
**Effects of a lack of equipoise**	**Health professional preferences and beliefs**: - Impact on patients - Treatment arms of the trial are so different	**Patient preferences reinforced by health professionals**—‘we can save your breast’ **Diagnostic journey doubts**

### Health professional themes

#### Influences on equipoise and recruitment—the treatment landscape has changed

The acceptability of TM as standard practice for MIBC changed during our study and especially following the international St Gallen consensus meeting in March 2017 [4, 5]. Despite guidelines recommending that patients should be encouraged to take part in well-designed RCTs, this consensus statement has inevitably influenced surgical opinion and increased confidence in breast conservation for MIBC.

Most of us take the view that although there’s no really hard evidence that conservation surgery for multifocal, multicentric [cancer] has been trialled properly, we know that the St. Gallen consensus is that it might be suitable, provided you can achieve clear margins. Because St. Gallen has pronounced on this then I think people are moving that way in terms of their threshold for doing conservation surgery. (Surgeon #3, Site C, non-recruiting site)

“Well there is no evidence from randomised control trials, but there is plenty of evidence that multiple cancer with clear margins has equal or similar survival and local recurrence rate, …. if you are going to believe large cohort evidence, then that’s an option for us (Surgeon #7, Site E, non-recruiting)

Many in the MDT seem convinced by the data that is emerging so far, even though that’s not trial data, there is no research data, especially when … they are truly multifocal, as opposed to multicentric I guess... So yeah, broadly multiple issues before we are actually at that stage that we randomise, but mostly I am sure it must be a shared issue with other centres. (Surgeon #16, Site B, recruiting site)

Consequentially treatment patterns at breast care centres changed with more BCS being offered as usual care. This, combined with the pool of eligible patients being smaller than expected, made it difficult to recruit patients to the trial.

When the evidence is coming out suggesting that breast conserving surgery and radiotherapy has a survival advantage over mastectomy, it’s quite hard to not offer that as an option for a patient, when you’re looking at somebody who has got a double E breast and you know perfectly well you could take 300 or 400 grams away, get both the tumours out and still leave them really nice breasts, very hard to say to them you need to have a mastectomy. (Surgeon #9, Site A, non-recruiting)

So I think probably the biggest problem was lack of patients suitable, so patients who had either got too extensive disease, and therefore wouldn’t have had conservation anyway, and that reduced the pool of patients down quite substantially… “the problem that we then had was lack of equipoise by the patient.” (Surgeon #3, Site C, non-recruiting)

#### The effects of lack of equipoise—health professional preferences and beliefs

***Conveying equipoise*** and running a trial within the context of shared decision-making consultations was challenging. Health professionals often felt uncomfortable conveying the current lack of evidence for treatment and did not present trial options in a balanced way because they were not in equipoise, believing that certain eligible patients might be more suitable for a breast conserving procedure.

It’s very difficult to sit there and say to the lady well the standard treatment would be this, but ….. we’ve now got to think of perhaps some different options, and at the moment we don’t know scientifically which is the better, to take all the breast tissue away or perhaps to be able to take just two areas away, and we’ve got a trial going on. I would say virtually everybody said well you said “you could preserve the breast beforehand, can you not do it now?” (Surgeon #9, Site A, non-recruiting)

The problem was with the surgeons … at the MDT, would say look this patient would be suitable for some kind of breast conserving procedure technically therefore they were not happy to randomise them to mastectomy. (Surgeon #6, Site J, recruiting)

I think it’s still the equipoise issue though particularly with this study. I know our clinicians are used to introducing studies with equipoise, but it’s cobbled with the fact that they’re only allowed to give limited information at a time when a patient normally asks questions about what the next treatment is going to be involving. (Nurse #11, Site D, recruiting)

And when you are selecting patients, a lot of patients … about a third of patients will say to me, “You tell me what the best operation for me is,” which is an almost impossible question to answer. (Surgeon #15, Site F, recruiting)

##### Impact on patients

Staff found discussing the RCT particularly challenging to talk to patients who expressed strong preferences and opinions for either treatment. These patients often had multiple contacts and received advice from a range of individuals during their pre-operative journey.

..either they wanted to save their breast, or they didn’t. Equipoise wasn’t coming into discussion, almost before you told them ‘you had breast cancer’ they knew what they wanted. (Surgeon #7, Site E, non-recruiting)

Women said “Well if you’re prepared to randomise me to breast conservation then that’s the thing I want, because you are not saying to me you have to have a mastectomy, you are saying I can go into this trial, therefore I am not having a mastectomy. (Surgeon #6, Site J, recruiting)

I think when they come to us they pretty much have made up their mind what is the treatment they want, and at that point we say well we don’t know what you’re going to get, you might get mastectomy even though they want the mammoplasty, then it’s something that puts them off, and that’s why the randomisation is the biggest barrier. (Nurse #13, Site B, recruiting)

I would sit in the MDT and I would sometimes highlight [MIAMI]… and then the patient was often discussed the same day that they are seen, so it was a difficult situation to then get in to see the surgeon… because they had often already been talked to by somebody else. (Nurse #14, Site K non-recruiting)

##### Treatment arms of the trial are so different

Staff also commented that an additional recruitment barrier was the notable difference in the surgical treatments, and consequently, it was a big decision for women to make.

I think that when it comes down to two treatments that are so different, it’s not like you’re choosing between one chemotherapy drug and another that may or may not have very slightly different side effects, we’re talking about two fundamentally different procedures with fundamentally different outcomes and I really do feel that a randomised trial was not the way forward to answer this question. As I say I am open to studies and things like that, but we needed to do the trial in a different way. So, I was expecting that it would be very difficult to recruit to. (Surgeon #15, Site F recruiting)

### Patient themes

#### Influences on recruitment—altruism and timing

##### Recruited patients: altruism

Patients recruited into the trial appreciated the detailed nature of trial information given to them and were generally satisfied with the questionnaires they completed. There was a common desire to help advance medical science for members of their family and the wider female population.

If there’s anything that can help women, I will do it. (P146, recruited, site F)

I have two grown up daughters and a daughter-in-law myself, and you want to do it for other people, you want to do it for the next generation. So that was the attitude I had when I accepted. (P569, recruited, site B)

Others agreed because they believed that taking part in the trial was the only way to receive BCS.

So, because there wouldn’t have been any other option, it would have been straight to mastectomy you see apart from the study. (P118, recruited, site D)

***Timing*** of study approach was crucial for all patients: One patient felt that being introduced to the trial during the initial consultation was too overwhelming, as she was still coming to terms with her diagnosis and had a fear of the cancer spreading.

You’re trying to process the fact that you’ve got what you’ve got, then you’re trying to process the fact that you’ve got to have a major operation at some point, and then all this stuff just gets thrown at you, and then the study as well. (P233, declined, site F)

Two participants were invited to take part in more than four research studies and questioned the burden of research.

There was a point where I began to think this is ridiculous perhaps they don’t know how many trials they are asking people to take part in. (P494, declined, site B)

#### Effects of lack of equipoise—patient preferences reinforced by health professionals

***We can save your breast*** Treatments available at breast care centres depended not only on a finding of multifocal cancer, but also upon which surgeon was present at the initial consultation when diagnostic biopsy results were given. Patients were frequently offered information about MIAMI after this consultation, which may have been too late for some patients. Several women had *a priori* treatment preferences (ahead of randomisation) relating to their age, fear of cancer spreading and the fact that cancer had been identified relatively early. Even so, two of them still agreed to take part and were randomised to their preferred treatment.

So they asked me if I would like to join the study and I said oh yeah I’ll try it, no problem…I wanted to have a full mastectomy, … it came back mastectomy and I went thank God… (P623, recruited, site J)

Proffered treatment options inadvertently discussed by surgeons during early consultations potentially risked undermining later discussions when RCT options were introduced with equipoise. Two (declining) women appeared to have been heavily influenced by the views and recommendations of their treating consultant. One was told, ‘we can save your breast’ and that a mammoplasty was ‘appropriate’.

He said, “We can save your breast if you would like to,” and I said, “Well if you can.” He would remove the cancerous lump and do a bit of reconstruction at the same time. So that was all agreed. …Then I was offered the MIAMI trial, [after the initial consultation] and the lady rang up and said to me, “[Doctor] has put you forward for the MIAMI trial.” Well now, I’d had this half an hour consultation … it had been planned with my consultant to have a lumpectomy… So that was the reason that I refused. (P494, declined, site B)

This [mammoplasty] obviously suited me, so at that time having to have a computer decide for me wasn’t an option. They told me what was best for myself, so that’s what I went with. (P542, declined, site B)

***Diagnostic journey doubts*** Patients reported that their opinions changed over time due to the influence of different advice from various individuals and improved understanding about treatments. Some women initially felt positive about joining MIAMI but found that ‘doubt began to set in’ as they progressed through their appointments. One woman acquired a preference for mammoplasty and was randomised to this treatment arm, another opted to have a mammoplasty after being randomised to the mastectomy arm.

So when they introduced MIAMI to me I was quite happy to have a look at it and go ahead with it… once I got into it doubt started to set in. (P569, recruited, site B)

..then a week or so after I thought I don’t know, and I will say from the day that they told me that’s what was going to happen right up until the morning that I went in to see the breast surgeon to confirm what was happening, I swayed between one or the other.… because it wasn’t so much the surgery it’s the reconstruction after it. (P146, recruited, site F)

## Discussion

This qualitative study embedded within the MIAMI surgical trial has illustrated the challenges of running a randomised controlled trial in a discipline where the evidence base for clinical decision-making changes rapidly and can be impacted by high profile international consensus meetings with dogmatic statements [4, 5]. Health professionals are very supportive of the need to provide sound evidence for surgical decisions through RCTs, but in practice, it can be extremely difficult to recruit participants. Strong patient and staff preferences for either of the two treatment options became apparent once MIAMI was open to recruitment, and health professionals struggled to communicate treatment options with equipoise. The scope for patients to choose their preferred treatment option in usual care meant that those unwilling to accept randomisation declined the trial. The randomisation process relinquishes control and increases the chance that they might not receive their preferred option. Altruism can often be a motivating factor for participation in RCTs where the main outcomes include the quality of life and allocated treatments are established surgical procedures [22]. Under these circumstances, women do not have any obvious treatment preferences. However, women involved with MIAMI expressed strong treatment preferences and were less agreeable to trial participation.

So is it possible to run RCTs in breast cancer surgery with two such different procedures? Timing of offering trial recruitment documentation, shared decision-making consultations, lack of equipoise and strong preferences for retaining or removing the breast influenced recruitment into the trial. Treatment decisions are made along the continuum of a complex patient pathway with challenges regarding the ideal time to introduce a trial to a patient. For the MIAMI trial, the RCT design was appropriate at the time of conception; however, things evolved (including implementation of the St Gallen statement) and were learnt during the trial recruitment process such that in retrospect this may not have been an appropriate trial design to answer this question.

Other surgical cancer trials have likewise struggled to recruit patients and have reported similar problems of patient preference for one arm of the trial, aversion to randomisation, too much information to read and lack of time in the clinic [23–25]. Medical trials have similar recruitment problems, and there is no evidence that surgical trials are more difficult to recruit into [26]. However, patient preference tends to have greater influence in surgical trials [27]. The need to ensure that recruiters are in equipoise has been well documented [28], with issues of patient preference and clinical equipoise likely to be more important where the difference between comparisons is greatest [29]. Nonetheless, innate conflict between routine clinical practice and recruitment to trials can be addressed through targeted interventions to understand and address recruitment issues, and close monitoring of the number of patients screened, eligible, approached and randomised [30, 31].

To improve upon weaker case-control study designs and evidence from expert opinion, which currently influence surgical practice [32], perhaps the way forward may be to raise the importance of well-designed ‘cohort multiple randomised controlled trials (cmRCT)’ or ‘trials within cohorts’ (TwiCs) with routinely collected quality of life measures [33, 34]. With these designs, intervention studies are performed within an observational longitudinal cohort as reported for the Dutch Utrecht cohort for multiple breast cancer intervention studies and long-term evaluation (UMBRELLA) [35]. Potential advantages include easier patient recruitment using a staged informed-consent procedure to improve generalisability and reduce contamination. However, robust pilot studies remain important to explore acceptance rates and compliance with any intervention.

The strength of this interview-based study includes gathering opinions from research nurses and surgeons at eight of the 10 trial sites (both recruiting and non-recruiting). Limitations include the small number of patients recruited to the trial and interviewed, although all recruited women were interviewed alongside some who declined the trial.

## Conclusions

Recruitment to breast cancer surgical trials presents significant barriers, especially where there is a substantial difference between the treatments offered and equipoise is not communicated effectively by recruiters. Patients can become overwhelmed by excessive requests for participation in research trials and inopportune timing of trial discussions. In future, funding for well-designed prospective cohort studies may be a more pragmatic way to generate high-quality evidence on which to base best practice in an era of patient-centred care.

## Data Availability

The anonymised data are available from the corresponding author on reasonable request.

## Abbreviations

IBTR: Ipsilateral breast tumour recurrence
MIAMI: Multiple Ipsilateral breast cancers for breAst conserving surgery versus Mastectomy
MIBC: Multiple ipsilateral breast cancer
RCT: Randomised controlled trial
cmRCT: Cohort multiple randomised controlled trials
TM: Therapeutic mammoplasty
TWiCs: Trials within cohorts

## References

[1] Winters ZE, Horsnell J, Elvers KT, Maxwell AJ, Jones LJ, Shaaban AM (2018). Systematic review of the impact of breast-conserving surgery on cancer outcomes of multiple ipsilateral breast cancers (MIBC). BJS Open..

[2] Winters ZE, Benson JR, on behalf of the MIAMI (Multiple Ipsilateral breast conserving surgery versus mastectomy) Trial Management Group (2018). Surgical treatment of multiple ipsilateral breast cancers. BJS..

[3] Boughey JC, Rosenkranz K, Nelson H (2012). Multiple ipsilateral breast cancers: can the breast be preserved?. Bull Am Coll Surg..

[4] Curigliano G, Burstein HJ, Winer EP, Gnant M, Dubsky P, Loibl S (2017). De-escalating and escalating treatments for early-stage breast cancer: the St. Gallen International Expert Consensus Conference on the Primary Therapy of Early Breast Cancer. Ann Oncol.

[5] Burstein HJ, Curigliano G, Loibl S, Dubsky P, Gnant M, Poortmans P (2019). Estimating the benefits of therapy for early-stage breast cancer: the St. Gallen International Consensus Guidelines for the primary therapy of early breast cancer. Ann Oncol..

[6] Bellavance EC, Kesmodel SB (2016). Decision-making in the surgical treatment of breast cancer: factors influencing women’s choices for mastectomy and breast conserving surgery. Front Oncol..

[7] Veronesi U, Cascinelli N, Mariani L, Greco M, Saccozzi R, Luini A (2002). Twenty-year follow up of a randomized study comparing breast conserving surgery with radical mastectomy for early breast cancer. N Engl J Med..

[8] Fisher B, Anderson S, Bryant J, Margolese RG, Deutsch M, Fisher ER (2002). Twenty-year follow up of a randomized trial comparing total mastectomy, lumpectomy, and lumpectomy plus irradiation for the treatment of invasive breast cancer. N Eng J Med..

[9] Early Breast Cancer Trialists Collaborative Group (EBCTCG). Effects of radiotherapy and of differences in the extent of surgery for early breast cancer on local recurrence and 15-year survival: an overview of the randomized trials. Lancet. 2005;366:2087–106. 10.1016/S0140-6736(05)67887-7 16360786

[10] Litiere S, Werutsky G, Fentiman IS, Rutgers E, Christiaens MR, van Limbergen E (2012). Breast-conserving therapy versus mastectomy for stage I-II breast cancer: 20-year follow up of the EORTC 10801 phase 3 randomised trial. Lancet..

[11] Benson JR (2012). Long-term outcome of breast conserving surgery. Lancet Oncol..

[12] Van Maaren M, de Munck L, de Bock GH, Jobsen JJ, van Dalen T, Linn SC (2016). 10-year survival after breast-conserving surgery plus radiotherapy compared with mastectomy in early breast cancer in the Netherlands. A population-based study. Lancet Oncol.

[13] Christiansen P, Carstensen SL, Ejlertsen B, Kroman N, Offersen B, Bodilsen A, Jensen MB (2018). Breast conserving surgery versus mastectomy: overall and relative survival-a population based study by the Danish Breast Cancer Cooperative Group (DBCG). Acta Oncol..

[14] de Boniface J, Frisell J, Bergkvist L, Andersson Y (2018). Breast-conserving surgery followed by whole-breast irradiation offers survival benefits over mastectomy without irradiation. BJS..

[15] Sinnadurai S, Kwong A, Hartman M, Tan EY, Bhoo-Pathy NT, Dahlui M (2018). Breast-conserving surgery *versus* mastectomy in young women with breast cancer in Asian settings. BJS Open..

[16] Gu J, Groot G, Boden C, Busch A, Holtslander L, Lim H (2018). Review of factors influencing women’s choice of mastectomy versus breast conserving therapy in early stage breast cancer: a systematic review. Clin Breast Cancer..

[17] Morrow M, Harris JR, Schnitt SJ (2012). Surgical margins in lumpectomy for breast cancer, bigger is not better. N Engl J Med..

[18] Benson JR, Jatoi I, Toi M (2019). Surgical management of multiple ipsilateral breast cancers. Future Oncol.

[19] Potter S, Trickey A, Rattay T, O’Connell RL, Dave R, Baker E (2020). Therapeutic mammaplasty is a safe and effective alternative to mastectomy with or without immediate breast reconstruction. BJS.

[20] Dicks E, Roome R, Chafe J, Powell E, McCrate F, Simmonds C, Etchegary H (2019). Factors influencing surgical treatment decisions for breast cancer: a qualitative exploration of surgeon and patient perspectives. Curr Oncol..

[21] Braun V, Clarke V (2006). Using thematic analysis in psychology. Qual Res Psychol.

[22] Bidad N, MacDonald L, Winters ZE, Edwards SJL, Emson M, Griffin CL (2016). How informed is declared altruism in clinical trials? A qualitative interview study of patient decision-making about the QUEST trials (Quality of Life after Mastectomy and Breast Reconstruction). Trials.

[23] Kaur G, Hutchison I, Mehanna H, Williamson P, Shaw R, Tudur Smith C. Barriers to recruitment for surgical trials in head and neck oncology: a survey of trial investigators. BMJ Open. 2013;3. 10.1136/bmjopen-2013-002625. 10.1136/bmjopen-2013-002625 PMC3641444 23585392

[24] Winters ZE, Emson M, Griffin C, Mills J, Hopwood P, Bidad N (2015). on behalf of the QUEST Trial Management Group. Learning from the QUEST multicentre feasibility randomization trials in breast reconstruction after mastectomy. BJS..

[25] Harrop E, Kelly J, Griffiths G, Casbard A, Nelson A (2016). Why do patients decline surgical trials? Findings from a qualitative interview study embedded in the Cancer Research UK BOLERO trial (Bladder cancer: Open versus Lapararoscopic or RObotic cystectomy). Trials..

[26] Cook JA, Ramsay CR, Norrie J (2008). Recruitment to publicly funded trials—are surgical trials really different?. Contemp Clin Trials..

[27] Ergina PL, Cook JA, Blazeby JM, Boutron I, Clavien PA, Reeves BC, Seiler CM, for the Balliol Collaboration (2009). Challenges in evaluating surgical innovation. Lancet.

[28] Donovan JL, Paramasivan S, de Salis I, Toerien M (2014). Clear obstacles and hidden challenges: understanding recruiter perspectives in six pragmatic randomised controlled trials. Trials..

[29] Paramasivan S, Huddart R, Hall E, Lewis R, Birtle A, Donovan JL (2011). Key issues in recruitment to randomised controlled trials with very different interventions: a qualitative investigation of recruitment to the SPARE trial (CRUK/07/011). Trials..

[30] Conefrey C, Donovan JL, Stein RC, Paramasivan S, Marshall A, Bartlett J (2020). OPTIMA Prelim Study Group. Strategies to improve recruitment to a de-escalation trial: a mixed-methods study of the OPTIMA Prelim Trial in Early Breast Cancer. Clin Oncol..

[31] Wilson C, Rooshenas L, Daisy PS, Elliott MJ, Strong S (2018). Development of a framework to improve the process of recruitment to randomised controlled trials (RCTs): the SEAR (Screened, Eligible, Approached, Randomised) framework. Trials.

[32] Cutress RI, McIntosh SA, Potter S, Goyal A, Kirwan CC, Harvey J (2018). on behalf of the Association of Breast Surgery Surgical Gap Analysis Working Group. Opportunities and priorities for breast surgical research. Lancet Oncol..

[33] Relton C, Torgerson D, O’Cathain A, Nichol J (2010). Rethinking pragmatic randomised controlled trials: introducing the “cohort multiple randomised controlled trial” design. BMJ..

[34] Relton C, Burbach M, Collett C, Flory J, Gerlich S, Holm S (2017). The ethics of ‘Trials within Cohorts’ (TwiCs): 2nd international symposium. Trials..

[35] Young-Afat DA, van Gils CH, van den Bongard HJGD, Verkooijen HM, on behalf of the UMBRELLA Study Group (2017). The Utrecht cohort for Multiple BREast cancer intervention studies and Long-term evaLuAtion (UMBRELLA): objectives, design, and baseline results. Breast Cancer Res Treat..

